# ﻿Soil-borne *Calonectria* (Hypocreales, Nectriaceae) associated with *Eucalyptus* plantations in Colombia

**DOI:** 10.3897/mycokeys.94.96301

**Published:** 2022-11-30

**Authors:** Nam Q. Pham, Seonju Marincowitz, ShuaiFei Chen, Carlos A. Rodas, Michael J. Wingfield

**Affiliations:** 1 Department of Plant and Soil Sciences, Forestry and Agricultural Biotechnology Institute (FABI), University of Pretoria, Pretoria, South Africa; 2 Department of Biochemistry, Genetics and Microbiology, Forestry and Agricultural Biotechnology Institute (FABI), University of Pretoria, Pretoria, South Africa; 3 Research Institute of Fast-growing Trees (RIFT), Chinese Academy of Forestry (CAF), Zhanjiang, Guangdong Province, China; 4 China Eucalypt Research Centre (CERC), Chinese Academy of Forestry (CAF), Zhanjiang, Guangdong Province, China; 5 Forestry Health Protection Programme, SmurfitKappa Colombia, Yumbo, Colombia

**Keywords:** Calonectria leaf and shoot blight, *Cylindrocladium*, multi-gene phylogeny, taxonomy, two new taxa

## Abstract

*Eucalyptus* spp. are widely planted in Colombia as an important component of a growing paper and pulp industry. Leaf and shoot blight caused by *Calonectria* spp. was one of the first disease problems to emerge in these plantations. A survey of *Eucalyptus* plantations in four forestry regions of Colombia during 2016 resulted in a large number of *Calonectria* isolates from soil samples collected in the understories of trees having symptoms of *Calonectria* leaf and shoot blight. The aim of this study was to identify and resolve the phylogenetic relationships for these isolates using DNA sequence comparisons of six gene regions as well as morphological characters. From a collection of 107 isolates, seven *Calonectria* species residing in three species complexes were identified. Two of these represented undescribed species, namely *C.exiguispora***sp. nov.** and *C.guahibo***sp. nov.***Calonectriaparvispora* and *C.spathulata* were the most commonly isolated species, each of which accounted for approximately 30% of the isolates. The results suggest that Colombia has a wide diversity of *Calonectria* spp. and that these could challenge *Eucalyptus* plantation forestry in the future.

## ﻿Introduction

Colombian plantation forestry is based primarily on non-native *Pinus* and *Eucalyptus* species, which have been widely deployed as an important component of the growing wood and paper industry. These plantations are based on short rotations, and in the case of *Eucalyptus*, clonal propagation has been established rapidly during the course of the last decade. There are currently approximately 540 000 ha of commercially managed plantations, of which *Eucalyptus* makes up a substantial component (20%) of this resource (MADR; https://www.minagricultura.gov.co/).

As plantation forestry has grown globally, damage due to insect pests and microbial pathogens has become increasingly important (Wingfield et al. 2008, 2015; Paine et al. 2011). Relevant diseases of planted *Eucalyptus* in Colombia include stem canker caused by species of Cryphonectriaceae and Botryosphaeriaceae (van der Merwe et al. 2001; Rodas et al. 2009), wilt and dieback caused by *Ceratocystisneglecta* (Rodas et al. 2008), Myrtle rust caused by *Austropucciniapsidii* (Rodas et al. 2015; Granados et al. 2017), as well as leaf and shoot blight caused by *Calonectria* species (Rodas et al. 2005). Of these, Calonectria leaf and shoot blight was amongst the first disease problems to emerge (Rodas et al. 2005; Rodas and Wingfield 2020).

Species of *Calonectria* (Hypocreales, Nectriaceae) have a wide distribution globally, especially in tropical and sub-tropical regions (Crous 2002; Lombard et al. 2010b; Marin-Felix et al. 2017). These fungi represent some of the most aggressive pathogens of agricultural, forestry, horticultural and ornamental plants (Crous 2002; Lombard et al. 2010b). *Calonectria* spp. are best known as root, shoot and foliar pathogens and can be associated with various disease symptoms, including damping-off, seedling blight, leaf and shoot blight, leaf spot, stem lesions, collar and root rot, fruit rot, and cutting rot (Sharma et al. 1984; Mohanan and Sharma 1985; Crous et al. 1991, 1998; Ferreira et al. 1995; Crous 2002; Old et al. 2003; Lombard et al. 2010b; Lopes et al. 2018).

In Colombia, the first outbreak of Calonectria leaf and shoot blight in *Eucalyptus* plantations occurred in 1998, where *Calonectriaspathulata* was shown to be the predominant pathogen (Rodas et al. 2005). High humidity and abundant free moisture in this region result in conditions highly conducive to disease outbreaks (Crous 2002; Rodas et al. 2005). Infections by *Calonectria* spp. have consequently resulted in severe defoliation and significant negative impacts on the growth of susceptible genotypes (Rodas et al. 2005).

*Calonectria* spp. are typically soil-borne fungi and many of these move between the soil environment and the leaf canopy of host trees (Crous 2002; Li et al. 2022). Previous studies of Calonectria leaf and shoot blight on *Eucalyptus* in Colombia considered only isolates from infected leaves (Rodas et al. 2005; Rodas and Wingfield 2020). In order to provide a more comprehensive overview of *Calonectria* species associated with *Eucalyptus* in Colombia, soil samples were collected from *Eucalyptus* plantations in Colombia, resulting in a large number of isolates. The aim of this study was to identify and resolve the phylogenetic relationships for these isolates using multi-gene DNA sequence comparisons as well as morphological characteristics.

## ﻿Materials and methods

### ﻿Sampling and fungal isolations

**Figure 1.**
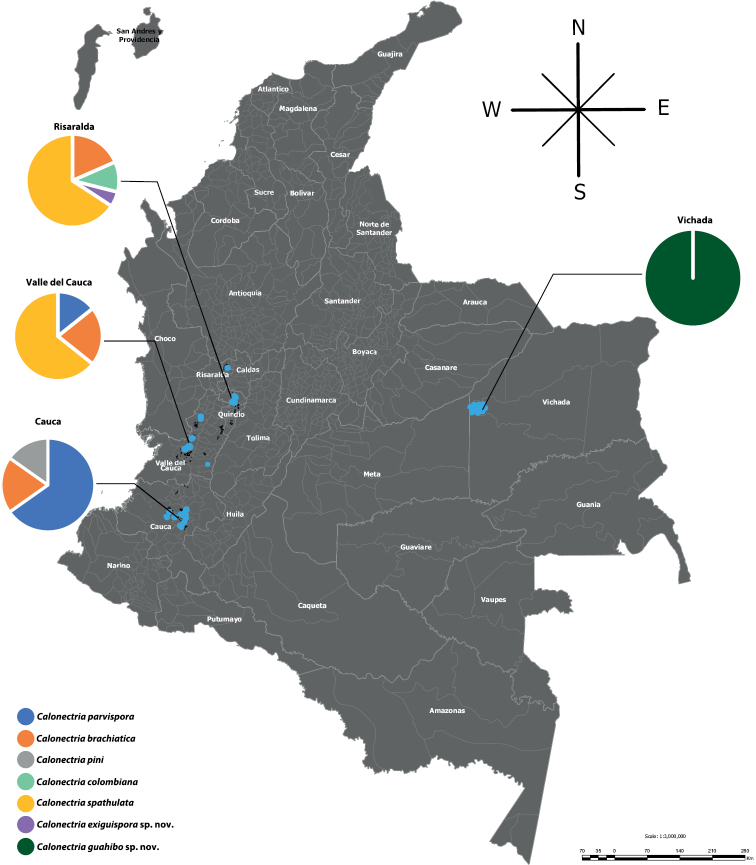
Geographic location of the sampling sites in Colombia, indicated as blue dots on the map, and the diversity of *Calonectria* spp. isolated from each region.

During 2016, surveys of *Eucalyptus* plantations were conducted in different forestry farms located across four provinces of Colombia, namely, Cauca, Risaralda, Valle del Cauca, and Vichada (Fig. 1; Suppl. material 2). Soil samples were taken in the understories of *Eucalyptus* trees having symptoms of Calonectria leaf and shoot blight. In addition, random soil samples were collected from the native vegetation surrounding the *Eucalyptus* plantations in these regions. Soils were packed in plastic bags and transferred to the laboratory for isolation. The samples were baited with germinating alfalfa (*Medicagosativa*) seeds following the method recommended by Crous (2002).

A dissection microscope was used to locate conidiophores and conidia typical of *Calonectria* on the infected alfalfa sprouts. These were lifted from the infected tissues using a sterile hypodermic needle and transferred to Petri dishes containing 2% (w/v) malt extract agar (MEA; 20 g malt extract, Biolab, Midrand, South Africa; 20 g Difco agar, Becton Dickinson, Maryland, USA; 1 L deionised water). Primary isolations were incubated for 3–7 d at 25 °C to allow fungal growth. Single hyphal tips were cut from the fungal colonies, transferred to fresh MEA plates, and incubated at 25 °C to obtain pure cultures. These cultures were deposited in the culture collection (CMW) of the Forestry and Agricultural Biotechnology Institute (FABI), University of Pretoria, South Africa. Representative cultures, including the ex-type strains of novel taxa, were deposited in the CMW-IA (the culture collection of Innovation Africa, University of Pretoria, Pretoria, South Africa). Dried-down specimens of sporulating cultures were deposited in the PRU (H.G.W.J. Schweickerdt Herbarium of the University of Pretoria, Pretoria, South Africa).

### ﻿DNA extraction, PCR amplification and sequencing

Prepman Ultra Sample Preparation Reagent (Thermo Fisher Scientific, Waltham, MA, USA) was used to extract the total genomic DNA from 7-d-old isolates grown on 2% MEA, following the manufacturer’s suggested protocols. A fragment of the actin (*ACT*), calmodulin (*CMDA*), histone H3 (*HIS3*), translation elongation factor 1-alpha (*TEF1*), β-tubulin (*TUB2*), and DNA‐directed RNA polymerase II second largest subunit (*RPB2*) gene regions were amplified using the primers ACT-512F and ACT-783R (Carbone and Kohn 1999), CAL-228F and CAL-2Rd (Carbone and Kohn 1999; Groenewald et al. 2013) CYLH3F and CYLH3R (Crous et al. 2004), EF1-728F and EF2 (O’Donnell and Cigelnik 1997; Carbone and Kohn 1999), T1 and CYLTUB1R (O’Donnell and Cigelnik 1997; Crous et al. 2004), and fRPB2‐5F and fRBP2‐7cR (Liu et al. 1999), respectively.

The PCR reactions and conditions were the same as those used by Pham et al. (2019) and Liu et al. (2020). ExoSAP-IT PCR Product Cleanup Reagent (Thermo Fisher Scientific, Waltham, MA, USA) was used to purify the Amplicons. Cleaned-up amplified fragments were sequenced in both directions using an ABI PRISM 3100 DNA sequencer (Thermo Fisher Scientific, Waltham, MA, USA) at the Sequencing Facility of the Faculty of Natural and Agricultural Sciences, University of Pretoria. Geneious Prime 2022.1.1 was used to assemble and edit the raw sequences (https://www.geneious.com). Sequences obtained in this study were deposited in GenBank (http://www.ncbi.nlm.nih.gov).

### ﻿Phylogenetic analyses

The sequences generated in this study were compared with those for previously published species of *Calonectria* sourced from the GenBank database (http://www.ncbi.nlm.nih.gov/) and subjected to phylogenetic analyses. Alignments of all sequences were assembled using the online version of MAFFT v. 7 (http://mafft.cbrc.jp/alignment/server/) (Katoh and Standley 2013) and then confirmed manually in MEGA v. 7 (Kumar et al. 2016). Maximum likelihood (ML) and Bayesian inference (BI) analyses were performed on data sets for each individual gene region and the combined data set. The most appropriate models were obtained using the software jModeltest v. 1.2.5. (Posada 2008). ML analyses were conducted using RaxML v. 8.2.4 on the CIPRES Science Gateway v. 3.3 (Stamatakis 2014) with a default GTR substitution matrix and 1,000 rapid bootstraps. BI analyses were performed using MrBayes v. 3.2.6 (Ronquist et al. 2012) on the CIPRES Science Gateway v. 3.3. Four Markov Chain Monte Carlo (MCMC) chains were run from a random starting tree for five million generations, and trees were sampled every 100^th^ generation. The first 25% of trees sampled were eliminated as burn‐in, and the remaining trees were used to determine the posterior probabilities. Sequences for two isolates (CBS 109167 and CBS 109168) of *Curvicladiellacignea* were used as the outgroup taxa in all phylogenetic analyses. Phylogenetic trees were viewed using MEGA v. 7 (Kumar et al. 2016).

### ﻿Morphology

The isolates were grown on synthetic nutrient-poor agar (SNA) (Nirenberg 1981) or together with alfalfa sprouts to induce the production of the asexual structures. Fruiting structures were initially mounted in water and replaced with 85% lactic acid for observation. Crosses between single hyphal tip isolates on minimal salt agar (MSA) were made to induce the production of a sexual state, as described by Pham et al. (2019). Nikon microscopes (Eclipse Ni, SMZ 18, Tokyo, Japan) were used to study the morphological characteristics. Images were captured using a Nikon DS-Ri2 camera mounted on the microscopes using the NIS-Elements BR program. Up to fifty measurements were made of all characteristic structures whenever possible. Dimensions were presented as minimum-maximum and with average ± standard deviation for the key morphological characteristics.

Colony characteristics were observed on 6-d and 30-d-old cultures on 2% MEA. Colours were described using the charts of Rayner (1970). Three replicates for each species were prepared to determine the optimum growth temperature. A mycelial plug (5 mm diam) from the margins of actively growing 4 d-old cultures was transferred to the centres of Petri dishes containing MEA. These cultures were grown at temperatures ranging from 5–35 °C at 5 °C intervals. Colony diameters perpendicular to each other were measured when colony growth reached the edges of Petri dishes at an optimum temperature, and averages were computed.

## ﻿Results

### ﻿Fungal isolates

**Figure 2.**
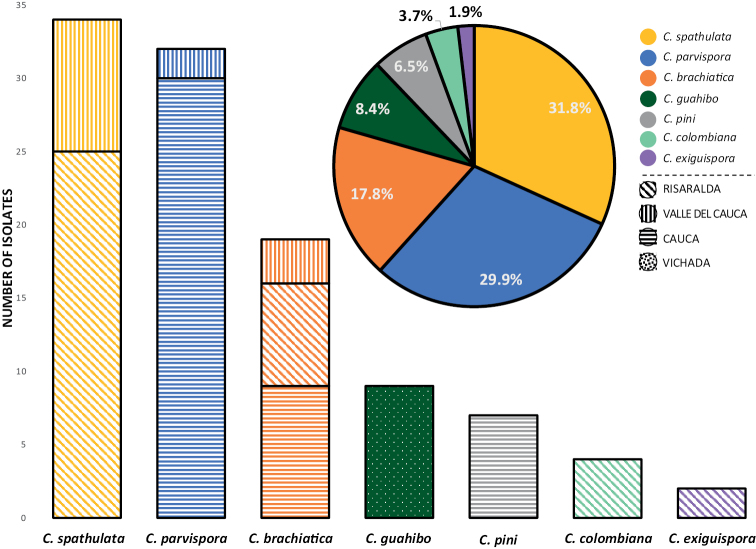
Relative occurrence of *Calonectria* species associated with *Eucalyptus* plantations in Colombia. Different species are represented by different colours. Isolates obtained from different regions are represented by different patterns in the bar chart.

A total of 107 isolates having morphological characteristics typical of *Calonectria* spp. were obtained from the soil samples (Suppl. material 2). Of these, 46 were from Cauca, 38 from Risaralda, 14 from Valle del Cauca, and nine from Vichada. Up to four different *Calonectria* spp. were detected in each of these regions (Figs 1, 2). Two of the most commonly isolated species each accounted for approximately 30% of the isolates (Fig. 2). The remaining isolates represented 1.9–17.8% of any one species (Fig. 2). All isolates were fast growing on SNA and MEA, producing abundant aerial mycelia, and scarce numbers of sclerotia, chlamydospores or fruiting structures in 3–4 w.

### ﻿Phylogenetic analyses

Sequence data were generated for all 107 isolates, which were approximately 250 bp for the *ACT* gene region, 660 bp for the *CMDA*, 430 bp for the *HIS3*, 1000 bp for the *RPB2*, 500 bp for the *TEF1*, and 560 bp for the *TUB2*. For the phylogenetic analyses of each individual data set, the HKY+G model was selected for *ACT*, the GTR+G model for *CMDA*, the GTR+G for *HIS3*, the TIM2ef+G for *RPB2*, the TPM1uf+G for *TUB2*, and the TPM3uf+I+G for *TEF1*. The ML tree for each individual gene region with bootstrap support values of ML and posterior probabilities of BI are presented in Suppl. material 1.

**Figure 3.**
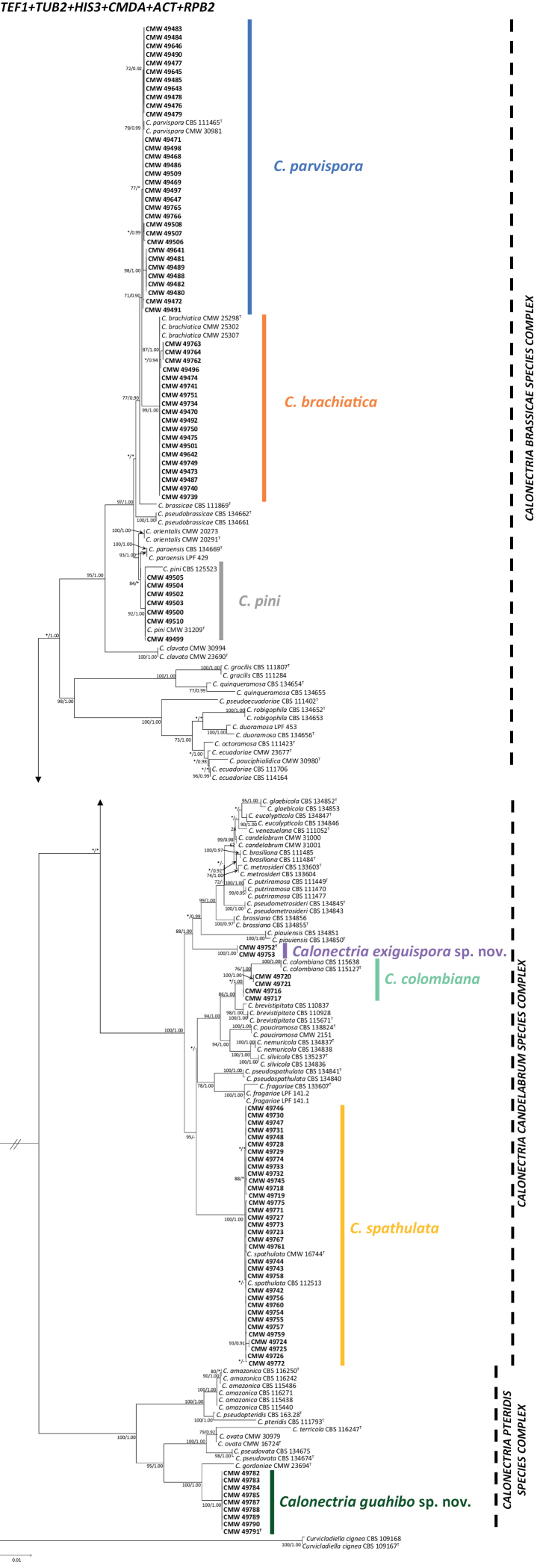
Phylogenetic tree based on maximum likelihood (ML) analysis of a combined DNA data set of *ACT*, *CMDA*, *HIS3*, *RPB2*, *TEF1* and *TUB2* sequences for *Calonectria* spp. Bootstrap values ≥ 70% for ML analyses and posterior probabilities values ≥ 0.90 obtained from Bayesian inference (BI) are indicated at the nodes as ML/BI. Bootstrap values < 70% or probabilities values < 0.90 are marked with “*”, and nodes lacking the support values are marked with “−”. Isolates representing ex-type material are marked with “T”. *Curvicladiellacignea* (isolate CBS 109167 and CBS 109168) represents the outgroup.

The combined sequence data set used in the phylogenetic analyses included 191 ingroup taxa and 3 315 characters, including alignment gaps. Concatenated sequence alignments of the six gene regions together with closely related *Calonectria* species were deposited in Zenodo (10.5281/zenodo.7195911). Topologies of the trees resulting from the ML and BI analyses were concordant and showed similar phylogenetic relationships between taxa. The ML tree with bootstrap support values for the ML and the posterior probabilities obtained from BI is presented in Fig. 3. Isolates considered in this study were all in the Prolate Group (Liu et al. 2020) and resided in either the *C.brassicae*, *C.candelabrum* or *C.pteridis* species complex.

The majority of the isolates resided in the *C.brassicae* species complex. Fifty-eight isolates in this complex clustered in three different clades. Of these, 32 isolates grouped in the same clade with the ex-type isolate of *C.parvispora*, 19 isolates clustered together with *C.brachiatica*, and seven with *C.pini*.

In the *C.candelabrum* species complex, 40 isolates clustered in three groups. Of these, four isolates grouped together with *C.colombiana*, 34 isolates with *C.spathulata*, and two isolates resided in a well-supported clade (ML/BI = 100/1.00) distinct from any known species in this complex and thus represent a novel taxon.

The remaining nine isolates residing in the *C.pteridis* species complex were closely related to *C.gordoniae* but formed an independent clade (ML/BI = 100/1.00) distinct from *C.gordoniae*, as well as other species in this complex. These isolates represent an undescribed taxon in *Calonectria*.

### ﻿Taxonomy

Based on phylogenetic analyses and morphological observations, isolates collected from soils in *Eucalyptus* plantations and their adjacent native vegetation in Colombia represented five previously described species, namely, *C.brachiatica*, *C.colombiana*, *C.parvispora*, *C.pini* and *C.spathulata*, and two novel species. One of these novel taxa resided in the *C.candelabrum* species complex and the other in the *C.pteridis* species complex. Descriptions for these species are provided as follows.

#### ﻿ Calonectria exiguispora

Taxon classificationFungiHypocrealesNectriaceae

N.Q. Pham, Marinc. & M.J. Wingf. sp. nov.

3DCC8EAD-8B6A-50B8-B7E6-8E2ADACBF40A

846456

Figs 4 , 6A, B

##### Etymology.

“exiguus” (Latin) = small + “spora” (Latin) = spores, referring to the small macroconidia produced by this species.

##### Diagnosis.

Phylogenetically close to *C.piauiensis* and *C.brassianae* but differs in having smaller macroconidia.

##### Type.

Colombia: Risaralda, Quinchía. Soils in *Eucalyptus* plantation. August 2016. C.A. Rodas. (***Holotype*** PRU(M) 4501, stored in a metabolically inactive state; ***ex*-*holotype*** CMW 49752, CMW-IA 160). GenBank: OP796405 (*ACT*); OP822275 (*CMDA*); OP822382 (*HIS3*); OP822489 (*RPB2*); OP822168 (*TEF1*); OP822596 (*TUB2*).

##### Description.

***Sexual morph*** not observed. ***Conidiophores*** scarce on SNA, consisting of conidiogenous apparatus and stipes, branched or simple. ***Stipes*** infrequent, elongated, septate, 75–273 µm long, 2–5 µm wide near base, tapering towards apex, simple or occasionally dichotomously branched, mostly being part of conidiogenous apparatus; ***vesicles*** terminal, slightly inflate to ellipsoidal, 2–5 µm wide. ***Conidiogenous apparatus*** hyaline, simple or branched in 1–3 (–4) tiers, uncommonly developing from stipes; ***main axis*** upright, septate, 20–275 × 3–7 µm; ***branches*** doliiform to cylindrical, primary branches 8–39 × 3–7 µm, secondary branches 8–24 × 2–6 µm, tertiary branches 10–23 × 2–5 µm, quarternary branches 10–14 × 3–4 µm. ***Conidiogenous cells*** holoblastic, hyaline, discrete, cylindrical to tapered above, often constricted near base, with periclinal thickening, 8–20 × 3–5 (11.8 ± 2.71 × 3.2 ± 0.5) µm. ***Macroconidia*** hyaline, cylindrical, round at apex, 1-septate, septum sub-median or median, guttulate, 21–40 × 3–4 (30.9 ± 4.09 × 3.5 ± 0.23) µm. ***Chlamydospores*** present, scarce, in clumps or in chains. ***Mega***- and ***microconidia*** not observed.

Colonies on 2% MEA in the dark for 6 d, white on surface, pale luteous in reverse, with moderate amount of aerial mycelium, with entire edges. Optimal growth temperature at 25 °C reaching 65.2 mm in 6 d, followed by 20 °C (57.3 mm), 15 °C (39.8 mm), 10 °C (19.7 mm), 5 °C (8.2 mm), no growth at 30 °C and 35 °C. Colonies kept at 30 °C and 35 °C being relocated to 25 °C for another 6 d revived (30 °C) and showed no growth (35 °C). Colonies on 2% MEA in the dark for 30 d, white to umber on surface, umber to dark brick in reverse, with flat mycelia.

##### Distribution.

Colombia.

##### Material examined.

Colombia: Risaralda, Quinchía. Soils in *Eucalyptus* sp. plantation. August 2016. C.A. Rodas. (PRU(M) 4502, stored in a metabolically inactive state; culture CMW 49753, CMW-IA 161).

##### Notes.

*Calonectriaexiguispora* is a member of the *C.candelabrum* species complex (Liu et al. 2020). It shares some characteristics with other species in the complex, such as 1-septate macroconidia and ‘ellipsoidal to obpyriform’ shape vesicle. However, it can be distinguished from most species in the complex by its smaller conidial dimensions (21–40 × 3–4 µm, avg. 30.9 × 3.5 µm) except for *C.brevistipitata* (29–35 × 3–4 µm, avg. 31 × 3.5 µm, isolated from Mexican soil) and *C.stipitate* (27–37 × 3–6, avg. 32 × 4 µm, isolated from Colombian *Eucalyptus* sp.) (Lombard et al. 2016). Nevertheless, these two species are distantly related to *C.exiguispora* (Fig. 3). Recently Liu et al. (2020) reduced *C.stipitata* to synonymy with *C.spathulata*, the conidial dimensions of which range from 48–100 × 4–6 µm (avg. 80 × 6 µm). They regarded the smaller conidial dimensions of *C. “stipitata*” as representing intraspecific variation. *Calonectriaexiguispora* is phylogenetically closely related to *C.piauiensis* and *C.brassianae*, which were isolated from soils associated with *Eucalyptusbrassiana* trees in Brazil (Alfenas et al. 2015). These two species, however, have much larger conidial dimensions: *C.piauiensis* (38–60 × 3–5 µm, avg. 49 × 4.5 µm) and *C.brassianae* (35–65 × 3–5 µm, avg. 53 × 4 µm) (Alfenas et al. 2015). It can be differentiated from its most closely related species by sequences of *ACT*, *CMDA*, *HIS3*, *RPB2*, *TEF1* and *TUB2* gene regions.

**Figure 4.**
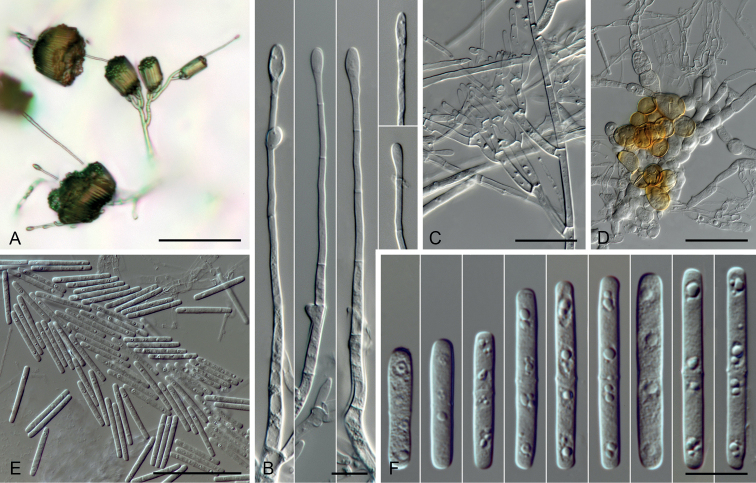
Micrographs of *Calonectriaexiguispora* sp. nov. (ex-holotype: CMW 49752 = CMW-IA 160). **A** conidiophores formed on SNA**B** stipes and vesicles **C** conidiogenous apparatus **D** chlamydospores **E** conidia (× 400) **F** conidia (×1 000). Scale bars: 100 µm (**A**); 50 µm (**D, E**); 25 µm (**C**); 10 µm (**B, F**).

#### ﻿ Calonectria guahibo

Taxon classificationFungiHypocrealesNectriaceae

N.Q. Pham, Marinc. & M.J. Wingf. sp. nov.

47C5B8B8-3ABD-5079-86AF-91F1467E87AD

846457

Figs 5 , 6E, F

##### Etymology.

Name refers to the indigenous people, Guahibo, native to Vichada, Colombia.

##### Diagnosis.

Closely related to *C.gordoniae* but differs in having smaller macroconidia.

##### Type.

Colombia: Vichada, Cumaribo. Soils in *Eucalyptus* plantation. August 2016. C.A. Rodas. (***Holotype*** PRU(M) 4503, stored in a metabolically inactive state; ***ex*-*holotype*** CMW 49791, CMW-IA 162). GenBank: OP796480 (*ACT*); OP822350 (*CMDA*); OP822457 (*HIS3*); OP822564 (*RPB2*); OP822243 (*TEF1*); OP822671 (*TUB2*).

##### Description.

***Sexual morph*** not observed. ***Conidiophores*** scarce on SNA, composed of conidiogenous apparatus and stipes. ***Stipes*** part of conidiogenous apparatus, elongated, septate, 81–223 µm long, 2–5 µm wide near base, tapering towards apex, simple, infrequently branched; ***vesicles*** slightly inflated to clavate, 2–5 µm wide. ***Conidiogenous apparatus*** hyaline, branched irregularly in 2–3 (–4) tiers; main axis upright, septate, 25–83 × 4–6 µm; ***branches*** doliiform to cylindrical, primary branches 11–23 × 4–6 µm, secondary branches 7–16 × 3–5 µm, tertiary branches 9–11 × 3–4 µm. ***Conidiogenous cells*** holoblastic, hyaline, discrete, cylindrical to ovoid, tapering towards apex, with perclinal thickening, 6–12 × 2–4 (9.3 ± 1.46 × 3.0 ± 0.52) µm. ***Macroconidia*** hyaline, cylindrical with round ends, 1-septate, straight, septum median or sub-median, 26–42 × 3–4 (31.7 ± 3.59 × 3.2 ± 0.19) µm. ***Chlamydospores*** present in clumps or in chains. ***Mega***- and ***microconidia*** not observed.

Colonies on 2% MEA after 6 d in the dark, growing circular, with fluffy aerial mycelia, above white to pale luteous towards centre, reverse luteous to umber towards centre. Optimal growth temperature at 30 °C reaching 61 mm, followed by 25 °C (57.5 mm), 20 °C (48.3 mm), 15 °C (21.8 mm), and no growth at 5, 10, and 35 °C. Colonies kept at 5, 10, and 35 °C revived after being relocated to 25 °C. Colonies on 2% MEA in the dark for 30 d, with cottony mycelia filled entire Petri dish, above saffron to umber with patches of white, reverse dark brick to sepia.

**Figure 5.**
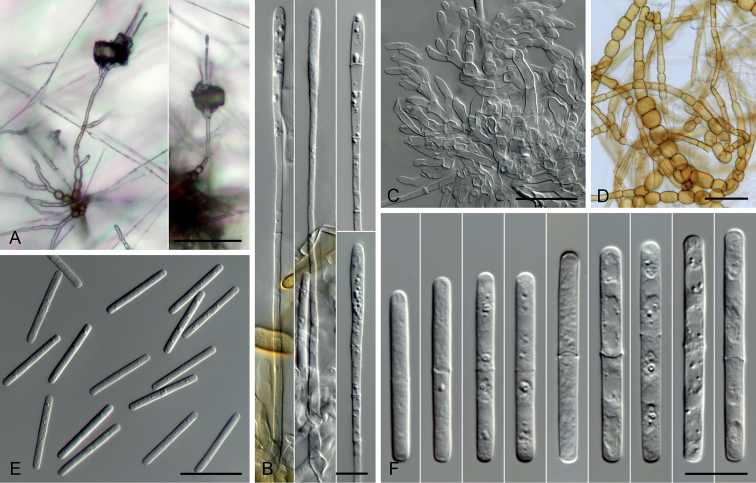
Micrographs of *Calonectriaguahibo* sp. nov. (ex-holotype: CMW 49791 = CMW-IA 162). **A** conidiophores formed on SNA**B** stipes and vesicles **C** conidiogenous apparatus **D** chlamydospores **E** conidia (× 400) **F** conidia (×1 000). Scale bars: 100 µm (**A**); 50 µm (**D**); 25 µm (**C, E**); 10 µm (**B, F**).

**Figure 6.**
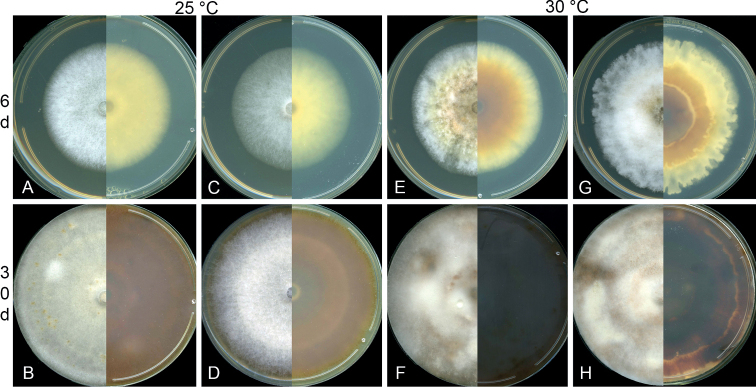
Culture morphology of *Calonectriaexiguispora* (**A–D**) at 25 °C and *C.guahibo* (**E–H**) at 30 °C in the dark for 6 d (**A, C, E, G**) and 30 d (**B, E, F, H**) at its optimum temperature **A, B** CMW 49752 (ex-holotype) **C, D** CMW 49753 **E, F** CMW 49791 (ex-holotype) **G, H** CMW 49782.

##### Distribution.

Colombia.

##### Material examined.

Colombia: Vichada, Cumaribo. Soils in *Eucalyptus* sp. plantation. August 2016. C.A. Rodas, *CMW 49782*.

##### Notes.

*Calonectriaguahibo* forms part of the *C.pteridis* species complex as a sister taxon to *C.gordoniae*. *Calonectriagordoniae* was reported from Florida, USA, causing leaf spots and blotches on loblolly bay (*Gordonialasianthus*) and is known to produce macroconidia (45–81 × 4–6 µm, avg. 61.7 × 5 µm) and microconidia (20–42 × 3–4 µm, avg. 32.5 × 3.6 µm) (Leahy et al. 2000). Leahy et al. (2000) reported slightly curved conidia which were not observed in *C.guahibo*. *Calonectriaguahibo* can be distinguished by its smaller conidia (26–42 × 3–4 µm, avg. 31.7 × 3.2 µm) from other closely related species, *i.e. C. ovata* (50–110 × 4–6 µm, avg. 70 × 5 µm) (Victor et al. 1997), *C.pseudovata* (55–50 × 4–7, avg. 69 × 5 µm) (Alfenas et al. 2015), and *C.terricola* (40–53 × 3–6 µm, avg. 46 × 4.5 µm) (Lombard et al. 2016). It can be differentiated from its most closely related species by sequences of *ACT*, *CMDA*, *HIS3*, *RPB2*, *TEF1* and *TUB2* gene regions.

## ﻿Discussion

A relatively large number of *Calonectria* species were discovered from soils collected in *Eucalyptus* plantations in four forestry regions of Colombia. All of the isolates were identified based on DNA sequence comparisons for six gene regions and supported by morphological characteristics. Seven species residing in three species complexes were identified. These include five previously described species, *C.brachiatica*, *C.parvispora* and *C.pini* in the *C.brassicae* species complex, and *C.colombiana* and *C.spathulata* in the *C.candelabrum* species complex and two novel taxa for which the names *C.exiguispora* and *C.guahibo* have been provided.

*Calonectriaparvispora* was one of the most commonly isolated species (29.9%) and was recovered from two forestry regions (Fig. 1). This species has previously been found in soils collected from Brazil and Colombia (Marin-Felix et al. 2017), but this is the first record of this species from soils associated with *Eucalyptus*. Interestingly, *C.brachiatica* and *C.pini* in the *C.brassicae* species complex were also found, which were previously isolated from *Pinus* cuttings displaying collar and root rot symptoms in Colombian nurseries (Lombard et al. 2009; Lombard et al. 2010a). *Calonectriapini* was previously collected in Valle da Cauca (Lombard et al. 2010a), and its appearance in this study suggests that it has a relatively wide distribution in Colombia.

*Calonectriaexiguispora*, described in this study, has extended the total number of species of the *C.candelabrum* species complex to 20 (Liu et al. 2020; Sanchez-Gonzalez et al. 2022). In addition, two previously described species in the *C.candelabrum* species complex, *C.colombiana* and *C.spathulata*, were also found. The latter species represented the majority of the isolates (31.8%). This is relevant because *C.spathulata* is a well-known pathogen commonly associated with leaf and shoot blight on *Eucalyptus*, and it has been reported from plantations in tropical regions of South America (Crous and Kang 2001; Crous 2002; Rodas et al. 2005). In this study, *C.spathulata* was also isolated from soils collected in natural rainforests surrounding *Eucalyptus* plantations in Risaralda, where the first outbreak of the disease occurred. It is possible that it is native to this area, but further studies, including those at a population genetics level, would be required to resolve that question.

*Calonectriaguahibo* represents a new addition to the *C.pteridis* species complex, which now includes eight species (Liu et al. 2020), all of which have 1-septate macroconidia and clavate or ovate vesicles. *Calonectriaguahibo* appears to have a limited distribution, with all isolates obtained from soils collected in plantations in Vichada, and interestingly, it was the only species found in this region. Although the *C.pteridis* species complex incorporates some of the most important pathogens of *Eucalyptus* (Crous 2002; Graça et al. 2009; Alfenas et al. 2013, 2015), nothing is known regarding the pathogenicity of the newly described *C.guahibo*.

Many previous reports of *Calonectria* spp. are considered to be of dubious significance because identifications were mostly based on morphology. It is now well-recognised that multi-gene markers together with morphological comparisons are required to identify these fungi with confidence. Consequently, this study has provided a more comprehensive understanding of the species diversity and distribution of *Calonectria* in Colombian *Eucalyptus* plantations. This should contribute to the establishment of an effective management strategy for the diseases caused by these fungi in plantations and nurseries.

Results of previous investigations and the present study have shown that soils associated with commercially propagated *Eucalyptus* spp. in tropical and subtropical regions represent a niche that is remarkably rich in species of *Calonectria* (Alfenas et al. 2015; Lombard et al. 2015; Li et al. 2017; Pham et al. 2019, 2022; Wu and Chen 2021). New species of these important fungi will most likely emerge when more extensive surveys are extended for the remaining areas in Colombia in the future. Further studies should also be conducted to determine the relative importance of the many *Calonectria* spp. residing in the soils associated with *Eucalyptus* plantations in the country.

## Supplementary Material

XML Treatment for Calonectria exiguispora

XML Treatment for Calonectria guahibo

## Supplementary materials

Supplementary material 1 Phylogenetic tree based on maximum likelihood (ML) analysis of individual gene region (ACT, CMDA, HIS3, TUB2, TEF1 and RBP2) Data type (word document) https://binary.pensoft.net/file/776036This dataset is made available under the Open Database License (http://opendatacommons.org/licenses/odbl/1.0/). The Open Database License (ODbL) is a license agreement intended to allow users to freely share, modify, and use this Dataset while maintaining this same freedom for others, provided that the original source and author(s) are credited.Nam Q. Pham, Seonju Marincowitz, ShuaiFei Chen, Carlos A. Rodas, Michael J. Wingfield

Supplementary material 2 Collection details and GenBank accessions of isolates included in the phylogenetic analyses Data type table (excel document) https://binary.pensoft.net/file/776037This dataset is made available under the Open Database License (http://opendatacommons.org/licenses/odbl/1.0/). The Open Database License (ODbL) is a license agreement intended to allow users to freely share, modify, and use this Dataset while maintaining this same freedom for others, provided that the original source and author(s) are credited.Nam Q. Pham, Seonju Marincowitz, ShuaiFei Chen, Carlos A. Rodas, Michael J. Wingfield
