# Supplementary material for: ﻿Soil-borne Calonectria (Hypocreales, Nectriaceae) associated with Eucalyptus plantations in Colombia
Source: MycoKeys. 2022 Nov 30;94:17–35. doi: 10.3897/mycokeys.94.96301 (PMC9836433; doi:10.3897/mycokeys.94.96301)
Supplement: Supplementary material 1 — Phylogenetic tree based on maximum likelihood (ML) analysis of individual gene region (ACT, CMDA, HIS3, TUB2, TEF1 and RBP2) [file mycokeys-94-017-s001.docx]

**Supplementary material 1**

**Figure S1.** Phylogenetic tree based on maximum likelihood (ML) analysis of a combined DNA data set of *ACT* sequences for *Calonectria* spp. Bootstrap values ≥70% for ML analyses and posterior probabilities values ≥0.90 obtained from Bayesian inference (BI) are indicated at the nodes as ML/BI. Bootstrap values <70% or probabilities values <0.90 are marked with “*”, and nodes lacking the support values are marked with “−”. Isolates representing ex-type material are marked with “T”. *Curvicladiella cignea* (isolate CBS 109167 and CBS 109168) represents the outgroup.

**Figure S2.** Phylogenetic tree based on maximum likelihood (ML) analysis of a combined DNA data set of *CMDA* sequences for *Calonectria* spp. Bootstrap values ≥70% for ML analyses and posterior probabilities values ≥0.90 obtained from Bayesian inference (BI) are indicated at the nodes as ML/BI. Bootstrap values <70% or probabilities values <0.90 are marked with “*”, and nodes lacking the support values are marked with “−”. Isolates representing ex-type material are marked with “T”. *Curvicladiella cignea* (isolate CBS 109167 and CBS 109168) represents the outgroup.

**Figure S3.** Phylogenetic tree based on maximum likelihood (ML) analysis of a combined DNA data set of *HIS3* sequences for *Calonectria* spp. Bootstrap values ≥70% for ML analyses and posterior probabilities values ≥0.90 obtained from Bayesian inference (BI) are indicated at the nodes as ML/BI. Bootstrap values <70% or probabilities values <0.90 are marked with “*”, and nodes lacking the support values are marked with “−”. Isolates representing ex-type material are marked with “T”. *Curvicladiella cignea* (isolate CBS 109167 and CBS 109168) represents the outgroup.

**Figure S4.** Phylogenetic tree based on maximum likelihood (ML) analysis of a combined DNA data set of *TUB2* sequences for *Calonectria* spp. Bootstrap values ≥70% for ML analyses and posterior probabilities values ≥0.90 obtained from Bayesian inference (BI) are indicated at the nodes as ML/BI. Bootstrap values <70% or probabilities values <0.90 are marked with “*”, and nodes lacking the support values are marked with “−”. Isolates representing ex-type material are marked with “T”. *Curvicladiella cignea* (isolate CBS 109167 and CBS 109168) represents the outgroup.

**Figure S5.** Phylogenetic tree based on maximum likelihood (ML) analysis of a combined DNA data set of *TEF1* sequences for *Calonectria* spp. Bootstrap values ≥70% for ML analyses and posterior probabilities values ≥0.90 obtained from Bayesian inference (BI) are indicated at the nodes as ML/BI. Bootstrap values <70% or probabilities values <0.90 are marked with “*”, and nodes lacking the support values are marked with “−”. Isolates representing ex-type material are marked with “T”. *Curvicladiella cignea* (isolate CBS 109167 and CBS 109168) represents the outgroup.

**Figure S6.** Phylogenetic tree based on maximum likelihood (ML) analysis of a combined DNA data set of *RPB2* sequences for *Calonectria* spp. Bootstrap values ≥70% for ML analyses and posterior probabilities values ≥0.90 obtained from Bayesian inference (BI) are indicated at the nodes as ML/BI. Bootstrap values <70% or probabilities values <0.90 are marked with “*”, and nodes lacking the support values are marked with “−”. Isolates representing ex-type material are marked with “T”. *Curvicladiella cignea* (isolate CBS 109167 and CBS 109168) represents the outgroup.

Note:

*Calonectria parvispora*

*Calonectria brachiatica*

*Calonectria pini*

*Calonectria exiguispora* sp. nov.

*Calonectria colombiana*

*Calonectria spathulata*

*Calonectria guahibo* sp. nov.

**Figure S1.**

**Figure S2.**

**Figure S3.**

**Figure S4.**

**Figure S5.**

**Figure S6.**
